# Bias

**DOI:** 10.1093/jhps/hnaf056

**Published:** 2025-09-25

**Authors:** Richard E Field

**Affiliations:** City St George’s, University of London

The word bias is thought to have entered the English language in the 1520s and was used to describe an ‘oblique or diagonal line’. It derives from the French word *biais*, meaning ‘a slant, a slope, an oblique’ [1].

The concept of bias in relation to scientific endeavour was explored by Francis Bacon (1561–1626) in his 1620 publication *Novum Organum* where he wrote ‘For what a man; had rather were true he more readily believes. Therefore, he rejects difficult things from impatience of research; sober things, because they narrow hope; the deeper things of nature, from superstition; the light of experience, from arrogance and pride, lest his mind should seem to be occupied with things mean and transitory; things not commonly believed, out of deference to the opinion of the vulgar’ [2].

In the enlightenment, David Hume (1711–76) and Immanuel Kant (1724–1804) wrote about observer bias and subjectivity. In the nineteenth century, the concepts of perception errors and illusions were discussed by Gustav Fechner and Wilhelm Wundt (1832–87). In the early twentieth century, Karl Pearson (1857–1936) and Ronald Fisher (1890–1962) introduced statistical analysis; defining bias ‘As systematic error in measurement’ [3, 4]. During the twentieth century, attention initially shifted to focus on memory errors, expectancy effects, and experimenter bias [5, 6] then to the influence of systemic cognitive biases due to heuristic thought processes [7] and bias that affects decision-making, finance, and policy [8, 9]. Since the turn of the century, researcher bias has been a topic of increased attention questioning the true value of publications [10] and demanding greater data transparency [11], with greater willingness of researchers to publish null results as well as studies that appear to demonstrate statistically significant results.

It would be foolhardy to claim that members of the hip preservation community are any less vulnerable to bias than other clinicians, whether they are physical therapists, hip arthroscopists, or peri-acetabular osteotomy (PAO) surgeons. There is no algorithm that will tell us how the interplay of hip morphology, tissue laxity, muscle development, and psychological factors determines which therapeutic strategy or combination of strategies will achieve the best clinical outcome for a particular patient. Each group has expertise in their therapeutic strategies and each may experience judgemental bias in their clinical assessments, treatment recommendations, and the interpretation of their outcomes.

After PAO surgery, many patients experience a period of uncertainty before they begin to enjoy the benefits of their intervention. For this reason, any reliable predictor of outcome will lessen stress for both patient and surgeon. In this issue, we present a study from Wudbhav Sankar and his team in Philadelphia [12] investigating the value of a pre-operative intra-articular corticosteroid injection (CSI) as a predictor for pain relief after PAO. The authors found that ≥ 60% pain relief from CSI was the optimal threshold for predicting a successful post-operative pain outcome. However, as only 13% of their patients failed to achieve > 75% improvement from surgery, they were unable to show that a poor response to CSI would be predictive of a poor surgical outcome. While the study does not provide a reliable predictor for the outcome of PAO, it is a valuable piece of the jigsaw that will be needed to generate a bias-free algorithm for patient treatment.

While appropriate patient selection and technically proficient PAO surgery remain our goals, it is inevitable that a proportion of PAO patients will experience persistent post-surgical pain. In this issue, we present a paper [13] from Niels Bang and his colleagues, in Denmark, to investigate whether the addition of 100 IU of type A Botulinum toxin (Botox) to a short-acting local anaesthetic (lidocaine 3 ml) offers prolonged analgesic benefit to patients who experience persistent pain after this intervention. The study was undertaken on a cohort of only 10 patients, with no placebo group for comparison. The results were variable, with only one study subject reporting the magnitude of pain reduction that might tempt clinicians to consider offering this treatment to their patients. The authors acknowledge these and other limitations to their study. However, they do conclude that ‘Botox has a promising and significant pain-reducing effect*’*. In support of this contention, larger studies on other chronic joint pain conditions have reported analgesic benefit using intra-articular Botulinum toxin A [14, 15] and a number of cellular mechanisms have been proposed to explain its action(s) [16]. It will be fascinating to see whether future Botox studies reveal null results or statistically significant therapeutic benefit for individuals with chronic post-surgical hip pain.

While the UK FASHIoN [17] and FAIT [18] studies are widely regarded as providing as close to bias-free comparisons of conservative and surgical treatment for femoro-acetabular impingement (FAI), one facet of the FAI debate now focuses on the management of cases with combined acetabular retroversion with femoral retro-torsion and the role of anteverting femoral and acetabular osteotomy as potential interventions to manage such cases. In this issue, we present a study co-ordinated by Kartik Logishetty, with clinical contributions from Truro, Sheffield, Ottawa, Reading, and London [19]. The authors have investigated the outcomes of isolated anteverting femoral or acetabular osteotomies and compared these interventions with a control group who underwent arthroscopic surgery. The study demonstrates that, for this subgroup of FAI patients, similar outcomes can be achieved by anteverting acetabular or anteverting femoral osteotomy. The value of an arthroscopically treated control group, without rotational deformity, may be questioned but this does provide a benchmark for the osteotomists to aim for.

We hope that you enjoy these and the other papers in this issue and that you will be favourably biased to choose the *Journal of Hip Preservation Surgery* for publication of your contributions to hip preservation surgery.

Conflict of interest: None declared.
